# Roux-en-Y Gastric Bypass in Obese Diabetic Rats Promotes Autophagy to Improve Lipid Metabolism through mTOR/p70S6K Signaling Pathway

**DOI:** 10.1155/2020/4326549

**Published:** 2020-03-26

**Authors:** Nanxi Ma, Rui Ma, Kaixin Tang, Xuesong Li, Bing He

**Affiliations:** Department of Endocrinology, Shengjing Hospital of China Medical University, Shenyang, Liaoning 110004, China

## Abstract

**Purpose:**

To investigate the effects of Roux-en-Y gastric bypass (RYGB) surgery on markers of liver mitochondrial dynamics and find new therapeutic basis on obese type 2 diabetes mellitus (T2DM) patients. *Materials and Methods*. Thirty-two rats were divided into nondiabetic group, diabetic group, sham group, and RYGB group. The Dual-energy X-ray absorptiometry (DEXA) was used to detect short-term *curriculum vitae* for rat body component and fat and lean mass. Hepatic lipid content and triglyceride levels were detected by Oil Red O staining. Western blotting was used to examine autophagy and mammalian target of rapamycin/P70S6 kinase (mTOR/p70S6K) pathway-related proteins. The carbon dioxide production from the oxidation of [^14^C] oleate was measured. Plasma glucose was measured by glucose oxidase assay. The insulin and C-peptide were detected. Triacylglyceride (TG) and free fat acid (FFA) in plasma were determined by enzymatic colorimetric assays.

**Results:**

RYGB improved metabolic parameters and enhanced plasma GLP-1 level, ameliorated the lipopexia, and increased insulin sensitivity in the liver; RYGB promoted the hepatic autophagy and inhibited the mTOR/p70S6K signaling pathway. GLP-1 reduced fat load and increased fatty acid *β*-oxidation by activated autophagy to regulate the hepatic lipid pathway through mTOR/p70S6K signaling pathway.

**Conclusions:**

RYGB may reduce liver lipid toxicity and improve insulin sensitivity through activating the hepatic fat hydrolysis pathway and inhibiting the liver fat synthesis pathway. However, the transport pathway of liver fat does not play a key role.

## 1. Introduction

Obesity is a disease characterized by abnormal or excessive fat accumulation that may impair health. It is a global health epidemic with more than 650 million people affected worldwide, according to the World Health Organization report. Research suggested that the prevalence rate of nonalcoholic fatty liver disease (NAFLD) is 2–44% in the general European population (including obese children) and 42.6–69.5% in people with type 2 diabetes [1]. Insulin-inhibiting lipolysis in obese T2DM patients promote fatty ectopic deposition and induces hepatic steatosis [2]. In China, the prevalence of NAFLD in overweight and obese patients with T2DM is 70% [3, 4]. NAFLD is a chronic liver disease characterized by hepatic fat deposition and is the pathological manifestation of metabolic syndrome in the liver [5]. The initial pathological change is simple steatosis, which can progress to liver fibrosis and liver cancer with increasing hepatotoxicity [6]. The reduction of liver fat deposition and increasing of liver insulin sensitivity could inhibit the occurrence and progression of NAFLD [7]. RYGB is a procedure in which doctors reduce the size of the stomach to a pouch about the size of an egg and attach it to the intestine. It is the most common surgical weight-loss procedure in the U.S. Roux-en-Y gastric bypass (RYGB) can effectively treat lipid metabolism disorders in obese T2DM patients, reduce hepatic lipid toxicity, improve liver insulin resistance, and delay or even reverse NAFLD [8, 9]. However, the mechanism that RYGB reduces the accumulation of liver fat is not yet clear.

Insulin resistance (IR) is the common pathogenesis of obesity, T2DM, and NAFLD, especially related to fat metabolism disorders [10]. Insulin resistance increases circulating FFA, which exceeds tissue compensatory capacity leading to fat ectopic deposition. The liver is an important organ in regulating lipid metabolism in the body, which mainly regulates hepatic lipid metabolism through fat synthesis, decomposition, and secretion pathways. This mechanism can avoid the accumulation of hepatic lipids and maintain local homeostasis in the liver [11]. During the insulin resistance, excessive FFA in the circulation is taken up by the liver, and the key enzyme of fatty synthesis diacylglycerol acyltransferase 2 (DGAT2) can synthesize TG on the basis of diacylglycerol using endogenous monounsaturated fatty acids [12]. Intrahepatic excess TG can be transported into the blood as a very low-density lipoprotein (VLDL) under the action of microsomal triglyceride transporter (MTP) [13]. And the excessive TG can be hydrolyzed into FFA and glycerol by hormone sensitive lipase (HSL) and adipose triacylglyceride lipase (ATGL); after that, FFA is transported into mitochondria or peroxisome to complete fatty acid oxidation [14]. As the rate of TG synthesis exceeds the rate of TG secretion and decomposition in the liver, the dynamic balance of hepatic lipid metabolism is broken, leading to the accumulation of TG locally in the liver, hepatic steatosis, and even liver fibrosis.

Autophagy is the process in which cells use lysosomes to degrade damaged organelles and macromolecules. Singh et al. found that autophagy can reduce intrahepatic fat content and relieve hepatic steatosis by hydrolyzing intracellular lipid droplets [15]. Rat hepatocyte primary cell was cultured with oleic acid or choline methionine-deficient culture medium and treated with 3-MA, phosphoinositide 3-kinase (PI3K) inhibitors, or siRNA of autophagy-related genes 5 (Atg5), autophagy inhibitor; then, intracellular TG was increased, the number of lipid droplets was increased, cell volume was enlarged, and the *β*-oxidation of fatty acid was reduced. Autophagy is an important pathway for lysosomal acid lipase to obtain TG [16]. During autophagy, the autophagy-related proteins form autophagosomes, which wrap the cytoplasmic lipid droplets and transfer to lysosomes. The phagocytic pathway of lysosome is involved in hepatic fat catabolism.

Our previous study suggested that the liver autophagy-related protein LC3-II/LC3-I levels increased as early as four weeks after RYGB, selective autophagy linker protein P62 decreased, autophagy activity was promoted, and intrahepatic fat accumulation decreased, suggesting that RYGB may reduce the hepatic lipid toxicity by upregulating autophagy activity [17]. In this study, we further investigated the changes of hepatic insulin sensitivity and fat synthesis, secretion, and catabolic pathway in obese T2DM rats at two, four, and eight weeks after RYGB; then, we focused on the mechanism of the reduction hepatic lipid toxicity, the improvement of liver insulin resistance, and the inhibition of NAFLD by RYGB.

## 2. Materials and Methods

### 2.1. Experimental Animals

Thirty-two Sprague-Dawley (SD) rats, four-week-old, male, 180–200 g were purchased from HFK Bioscience Co., Ltd., Beijing, China [18]. All animals were housed in individual cages under controlled temperature (24 ± 2°C) and humidity in a 12 h light/dark cycle. Standard pelleted chow and drinking water were available *ad libitum*. The rats were allowed to acclimate to these conditions for at least one week. Rats were randomly assigned to four groups, the nondiabetic group received a standardized diet (18% fat, 25% protein, and 57% carbohydrate), while the diabetic group, diabetic sham RYGB group, and RYGB group received a high-fat diet (40% fat, 18% protein, and 42% carbohydrate) for six weeks [17]. A low dose of STZ (streptozocin, 25 mg/kg, Sigma-Aldrich, St Louis, MO, USA) was then injected through the tail vein in the model group at the end of six weeks [17]. The same dose of buffer, 0.1 mol/l citric acid, was used in the control group. Rats with nonfasting blood glucose ≥ 16.7 mmol/l [19] for four weeks were considered to be the diabetic. Then, rats were randomly assigned to the diabetic (*n* = 8), diabetic sham (*n* = 8), and diabetic RYGB group (*n* = 8). All procedures were approved by Animal Care and Use Committee of Shengjing Hospital of China Medical University.

### 2.2. Animal and Animal Model Preparation

The rats were fasted for 24 h preoperatively for gastrointestinal surgery which were anesthetized with an intraperitoneal injection of 1% sodium pentobarbital solution (5 ml/kg). In the diabetic RYGB group, the distal stomach was closed through double ligation with 2-0 silk yarn to create an ~20% gastric pouch; the small intestine was transected to produce a 15 cm biliopancreatic limb, a 10 cm alimentary (Roux) limb, and a 33 cm common channel. Gastrojejunal and jejunojejunostomies were carried out using interrupted 5-0 silk sutures, and the muscle layer and skin were closed using 4-0 silk. Rats in the sham operation group received similar preoperative or postoperative care as RYGB rats. The incision in the gastrointestinal tract was performed as RYGB rats; however, the incision was reanastomosed at the original transection site. After surgery, rats were given approximately ~20 ml/kg saline subcutaneously to prevent dehydration, then administrated with 1 mg/kg meloxicam via subcutaneous injection every 8 h for the first 24 h [18]. Rats were housed individually, and the weight, food intake rate, and blood glucose of rats were measured every week. At eight weeks after surgery, all rats were euthanized, and the liver tissues were collected and stored at −80°C for further analysis.

### 2.3. Hyperinsulinemic–Euglycemic Clamp

After fasting, rats were anesthetized, the catheter was placed, and the right internal jugular vein was implanted for infusion and left carotid artery for blood collection, as previously described [20]. As rats were fasted for twelve hours, insulin (Novolin R, Novo Nordisk Pharmaceuticals) and 20% glucose were injected through the jugular vein catheter. Arterial blood glucose was measured every five minutes, and glucose infusion rate was adjusted to maintain blood glucose concentration stability. [6-^3^H] glucose (20 *μ*Ci bolus + 0.4 *μ*Ci/min infusion) was administrated to assess endogenous glucose production. The hepatic insulin sensitivity (IS) was showed as the hepatic insulin sensitivity index (HISI), HISI = 1/(hepatic glucose production∗fasting insulin).

### 2.4. Whole-Body Composition

The dual-energy X-ray absorptiometry (DEXA, GE Lunar Prodigy) was used, and an internal standard was adapted for rat measurements. DEXA was suitable for the determination of whole-body composition in small laboratory animals by directly measuring fat, fat-free, and mineral bone masses [21]. All rats were fasted and sedated before scanning. Short-term *curriculum vitae* for each rat body component were estimated after the end of the scan. Fat and lean mass of rats were determined weekly after surgery.

### 2.5. Liver Tissue Lipid Content

Oil Red O staining was performed [22]. Samples were fixed in 3% formaldehyde overnight, excess formaldehyde was removed by three rinses in deionized water for 30 s, and then samples were embedded in Tissue-Tek optimal cutting temperature compound (Sakura Finetek) and sectioned (7 *μ*m) for neutral lipid staining using Oil Red O. Quantification of hepatocytes size and Oil Red O-stained area was performed by the ImageJ software. Sections were photographed at X400 (Oil Red O) magnification.

Hepatic triglyceride levels were measured using a colorimetric kit (Nanjing Jiancheng Institute of Bioengineering Institute, China). Liver tissue was extracted from frozen, fundamental tissue by mechanical homogenization in 10× volume of anhydrous ethanolunder ice bath conditions. The organic and aqueous phases were removed, and samples were centrifugated at 2,500 rpm for 10 min. A small aliquot (10–30 *μ*l) was removed, and the triglyceride concentrations were determined.

### 2.6. Western Blotting

The protein concentration was measured with Pierce™ BCA Protein Assay Kit (Thermo Fisher Scientific Inc., Rockford, IL). Aliquots containing 20 mg protein samples were separated by 8%-12% SDS-polyacrylamide gel. Then, the membranes were immunoblotted separately with primary antibody against DGAT2 (1 : 1000, Novus Biologicals), p-HSL (1 : 1000, Cell Signaling Technology), ATGL (1 : 1000, Bio-Techne), MTP (1 : 1000, Absin), LC3A/B (1 : 1000, Cell Signaling Technology), P62 (1 : 1000, Cell Signaling Technology), mTOR (1 : 1000, Cell Signaling Technology), p-mTOR (1 : 1000, Cell Signaling Technology), p70S6K (1 : 1000, Abcam), p-p70S6K (1 : 1000, Abcam), and GAPDH (1 : 2000, Proteintech) antibodies at 4°C for overnight. Protein bands were assessed using ECL solution (Beyotime, China) and detected by Image Quant LAS-4000 mini (GE, USA). The band intensity was assessed with GELPRO32.

### 2.7. Cells and Cell Culture

HepG2 (HB-8065) cells were purchased from ACTT and maintained at 37°C in DMEM containing 10% FBS in a 5% CO_2_ atmosphere. Cells were pretreated with 50 *μ*M chloroquine (Sigma) or 1 *μ*M MHY1485 (Med Chem Express), which is a mTOR agonist [23]. Cells were treated with 125 mM oleate for 24 h; then, medium were changed by supplement with 10 nM exendin-4 (Sigma) to maintain the fatty acids stress for 24 h. After that, HepG2 cells were cultured without exendin-4 for an additional 24 h. After treatment with exendin-4 and oleate or oleate alone for 24 h, cells were harvested.

### 2.8. Fluorescence Microscopy

Cells were fixed with 3% paraformaldehyde, blocked, and incubated with the primary and corresponding Cy5 and antihuman IgG (Texas Red™ Conjugated) secondary antibody. Lipid droplets were stained by incubating cells with BODIPY 493/503 (Invitrogen) for 30 min, fixed, and processed for immunofluorescence as described previous [24].

### 2.9. Fatty Acid *β*-Oxidation Assay

The rate of carbon dioxide production from the oxidation of [^14^C] oleate was measured. Cells were cultured in the presence of [^14^C] oleate-BSA complex, and the released [^14^C] carbon dioxide was trapped for 1 h at 37°C onto filter paper soaked in 100 mM sodium hydroxide. The rate of *β*-oxidation was calculated as the speed of trapped [^14^C] carbon dioxide produced.

### 2.10. Experimental Methods

Plasma glucose was measured with the glucose oxidase method (Breez 2, Germany). The concentration of insulin and C-peptide in the plasma was determined using enzyme-linked immunosorbent assay (ELISA) kits (USCN, Wuhan, China). TG and FFA concentrations in plasma were determined using enzymatic colorimetric assays (Nanjing Jiancheng Bioengineering Institute, Nanjing, China) according to the manufacturer's protocol.

### 2.11. Statistical Analysis

SPSS version 21.0 was used for the statistical analysis, and the quantitative data were presented as mean ± standard deviation (SD). The two-way ANOVA with Tukey test were used in order to compare results among groups. *P* < 0.05 was considered to be statistically significant.

## 3. Results

### 3.1. RYGB Improved Metabolic Parameters and Enhanced Plasma GLP-1 Level

**Figure 1:**
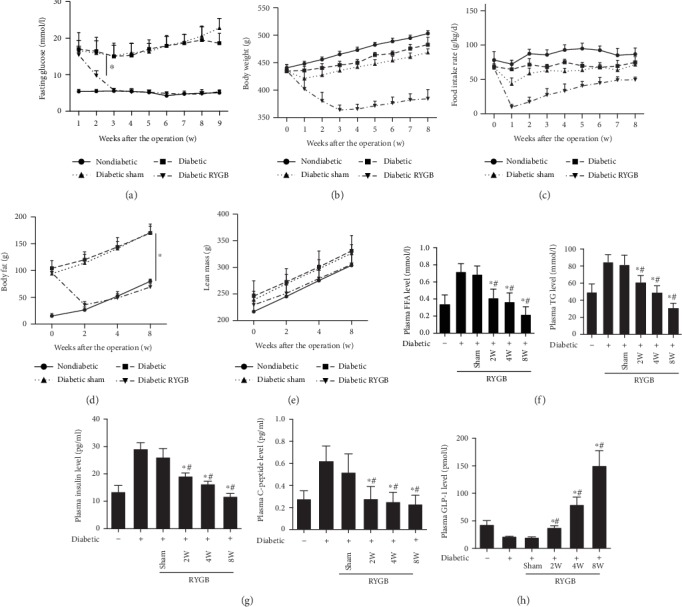
RYGB improved metabolic parameters and enhanced plasma GLP-1 level (*n* = 8). (a) Fasting blood glucose levels, (b) body weight, (c) food intake rate, (d) body fat, (e) lean mass, (f) plasma FFA and TG level, and (g) the levels of insulin and C-peptide. And the sham group was day 0 of RYGB group. All results are expressed as the mean ± SD. ^∗^Means *P* < 0.05 between diabetic group; ^#^means *P* < 0.05 between diabetic sham group.

Fasting blood glucose levels were perpetually reduced at two weeks (*P* < 0.05) and close to the nondiabetic group at three weeks after the RYGB operation and then remained stable. Rats in sham group did not show the significant changes (Figure 1(a)). Two weeks after the operation, rats in RYGB group showed 14% weight loss and achieved its lowest point in the third week post RYGB and then rebounded slightly and maintained steady state until eight weeks postoperatively (Figure 1(b)). At one week after surgery, food intake rate decreased in rats from the diabetic RYGB and diabetic sham groups, but there was no difference in food intake rate between the diabetic sham rats and untreated rats two weeks after surgery (Figure 1(c)). To examine the effects of RYGB on body composition after surgery, we performed DEXA analysis in each of the groups. The body fat in the diabetic and diabetic sham groups was markedly increased compared with that in rats of the nondiabetic group, and the decrease was dramatic at eight weeks after RYGB surgery (*P* < 0.05) (Figure 1(d)). However, there was no significant difference in lean mass between the groups (Figure 1(e)). These results suggested that the reduction in weight post RYGB is mainly caused by the decrease of body fat.

Moreover, RYGB also had a significant effect on the plasma lipid profile. Compared with the sham group, the concentration of FFA and TG decreased in RYGB group at two weeks post RYGB (*P* < 0.05) (Figure 1(f)). The levels of insulin and C-peptide in RYGB group were significantly lower than that of sham and diabetic groups (*P* < 0.05) (Figure 1(g)). Moreover, GLP-1 was significantly increased in the diabetic RYGB group than that in the diabetic and sham groups (*P* < 0.05) (Figure 1(h)).

### 3.2. RYGB Ameliorated the Lipopexia and Increased Insulin Sensitivity in the Liver

**Figure 2:**
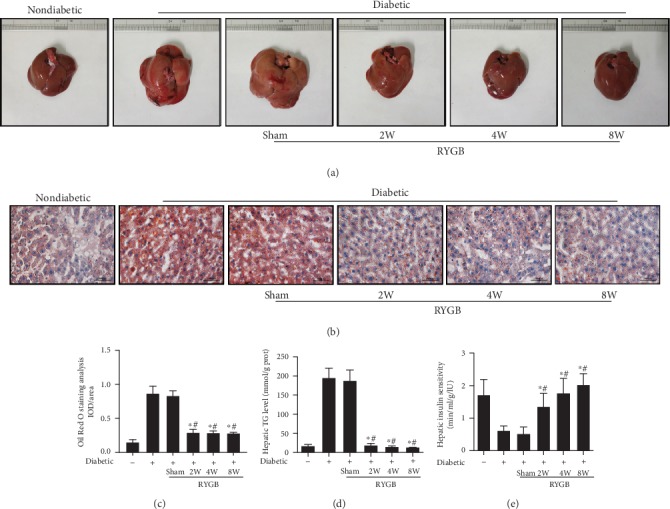
RYGB ameliorated the lipopexia and increased insulin sensitivity in the liver (*n* = 8). (a and b) Hepatic samples of each group, (c) the liver stained for Oil Red O (scale bar = 50 *μ*m), (d) hepatic triglyceride content, and (e) hepatic insulin sensitivity. And the sham group was day 0 of RYGB group. All results are expressed as the mean ± SD. ^∗^Means *P* < 0.05 between diabetic group; ^#^means *P* < 0.05 between diabetic sham group.

We collected hepatic samples of each group and found that livers in diabetic sham groups were brownish yellow with rounded and obtuse margins whereas the liver fat mass in the RYGB group dramatic declined (Figure 2(a)). The liver stained for Oil Red O showed the same results (Figures 2(b) and 2(c)). The diabetic RYGB rats had significantly reduced percent positive staining for neutral lipid and reduced lipid droplet size and number as indicated by Oil Red O staining compared with the diabetic and diabetic sham rats. In accordance, compared to the diabetic sham group, hepatic triglyceride content was significantly reduced in diabetic RYGB rats (*P* < 0.05). There was no difference between diabetic group and diabetic sham group (*P* > 0.05) (Figure 2(d)).

The hepatic insulin sensitivity was examined. HISI in the diabetic RYGB group was significantly increased compared with that in the diabetic sham group (*P* < 0.05) (Figure 2(e)). These results suggested that RYGB efficiently improved hepatic insulin resistance in obese T2DM rats.

### 3.3. Effects of RYGB on the Hepatic Lipid Pathway

**Figure 3:**
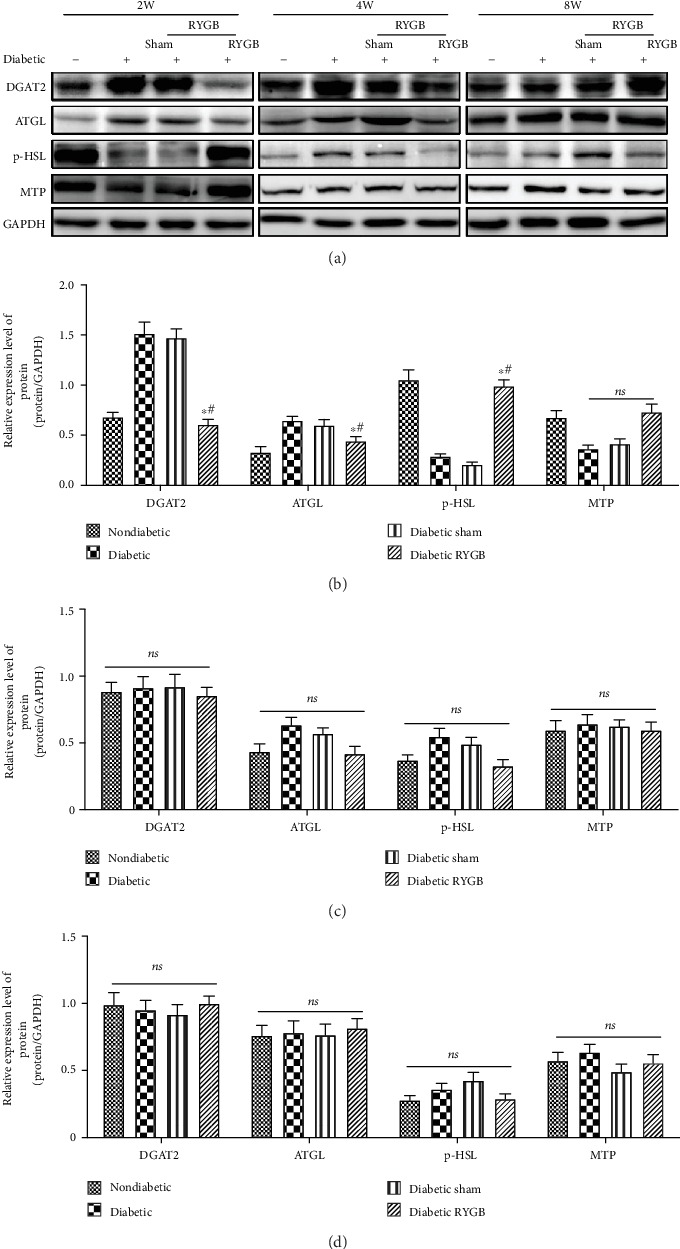
Effects of RYGB on the hepatic lipid pathway (*n* = 8). (a) Western blot, (b) the bar graph of the expression levels of key enzymes in hepatic lipid metabolism pathway two weeks postoperative, (c) the bar graph of the expression levels of key enzymes in hepatic lipid metabolism pathway four weeks postoperative, and (d) the bar graph of the expression levels of key enzymes in hepatic lipid metabolism pathway eight weeks postoperative. All results are expressed as the mean ± SD. ^∗^Means *P* < 0.05 between diabetic group; ^#^means *P* < 0.05 between diabetic sham group.

We examined the effects of RYGB on the expression levels of key enzymes in hepatic lipid metabolism pathway. Western blot showed that at two weeks after operation, the expression of DGAT2 and ATGL decreased significantly in the RYGB group compared with the sham group, while p-HSL increased significantly (*P* < 0.05) (Figures 3(a) and 3(b)). In the secretory pathway of hepatic lipid metabolism, the expression level of MTP was increased in the RYGB group, although there were no significant differences between the groups (*P* > 0.05) (Figures 3(a) and 3(b)). These results showed that RYGB could improve the hepatic lipid accumulation in short term by restrained triglyceride synthesis and facilitated hydrolase activity. Moreover, no significant difference was detected in the key enzymes in each metabolic pathway between the groups at four (Figures 3(a) and 3(c)) and eight weeks after surgery (Figures 3(a) and 3(d)). These results suggested that the key enzymes in hepatic lipid metabolism may not take effect in maintaining long-term hepatic lipid homeostasis after surgery.

### 3.4. RYGB Promoted the Hepatic Autophagy

**Figure 4:**
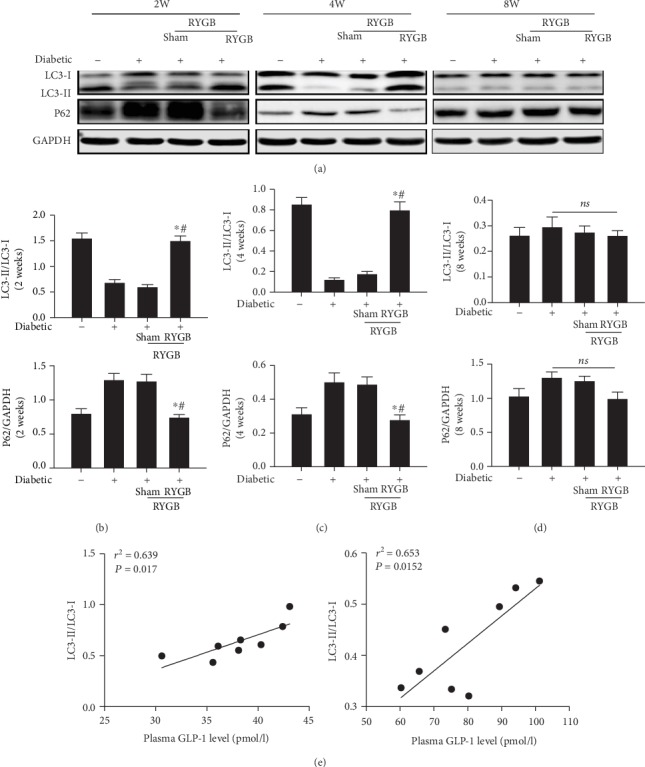
RYGB promoted the hepatic autophagy (*n* = 8). (a) Western blot, (b) the bar graph of the hepatic autophagy-related protein of two weeks, (c) the bar graph of the hepatic autophagy-related protein of four weeks, (d) the bar graph of the hepatic autophagy-related protein of eight weeks, (e) the association between the plasma GLP-1 content and the hepatic autophagy markers in two weeks after RYGB, and (f) the association between the plasma GLP-1 content and the hepatic autophagy markers in four weeks after RYGB. All results are expressed as the mean ± SD. ^∗^Means *P* < 0.05 between diabetic group; ^#^means *P* < 0.05 between diabetic sham group.

Autophagy plays a critical role in lipid metabolism since it shuttles lipid droplets to the lysosome where they are hydrolyzed by lysosomal lipase into FFA and glycerol [25]. The hepatic autophagy was examined to identify the mechanism that RYGB reduces lipid stores in liver tissue. Compared with sham group, the ratio of LC3 II/LC3 I was increased significantly, and the expression of P62 was dramatic declined (*P* < 0.05) at two weeks after RYGB (Figures 4(a)to 4(b)). At four weeks after operation, the expression of autophagy-related protein LC3 II/I was significantly increased, and the expression of P62 (*P* < 0.05) was decreased in the RYGB group (Figures 4(a) and 4(c)). At eight weeks after operation, the expression of autophagy-related protein had no significant differences between the groups (*P* > 0.05) (Figures 4(a) and 4(d)). According to the experimental results of Western blot, it was confirmed that RYGB significantly stimulated the activity of autophagy. These results showed that the increase of autophagy played a pivotal role in the short-term and long-term relief of hepatic lipid toxicity and lipid metabolism disturbance after RYGB. Furthermore, under the multiple linear regression analysis, we also found a strong positive association between the plasma GLP-1 content and the hepatic autophagy markers: LC3 II/I (*r*^2^ = 0.639, *P* = 0.017) in two weeks and LC3 II/I (*r*^2^ = 0.653, *P* = 0.0152) in four weeks after RYGB (Figure 4(e)).

### 3.5. RYGB Inhibits the mTOR/p70S6K Signaling Pathway

**Figure 5:**
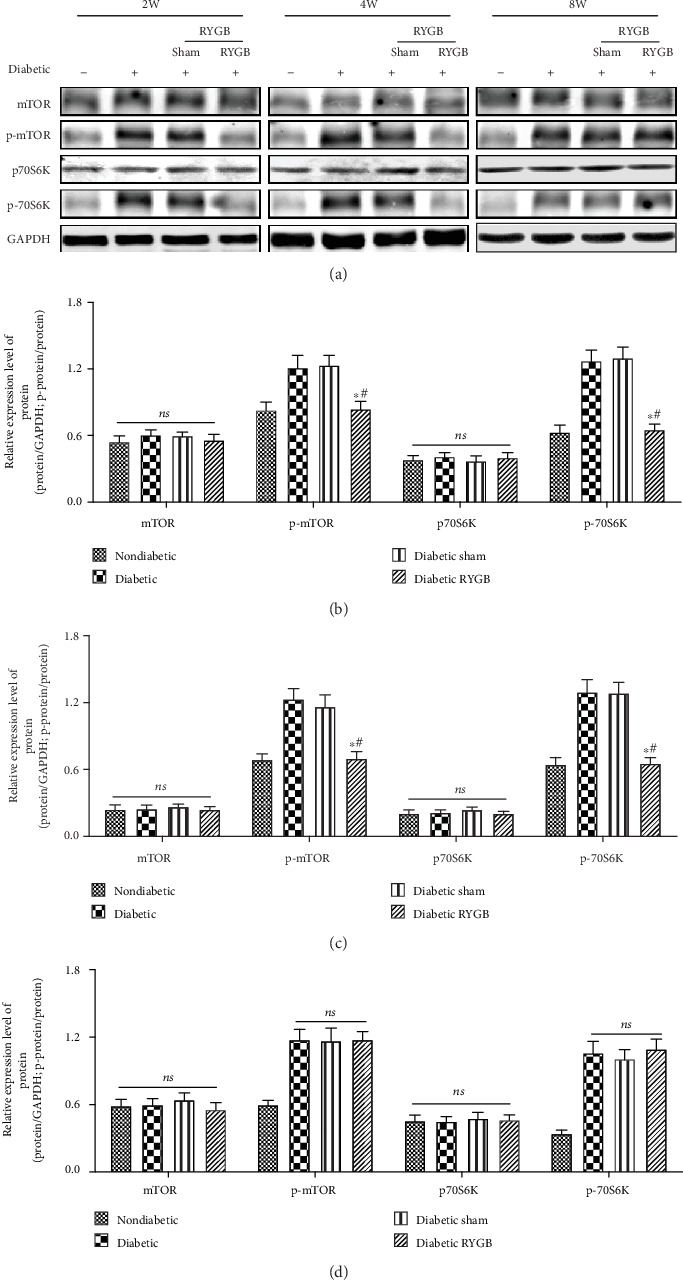
RYGB inhibits the mTOR/p70S6K signaling pathway (*n* = 8). (a) Western blot, (b) the bar graph of the expression levels of the mTOR/p70S6K signaling pathway of two weeks, (c) the bar graph of the expression levels of the mTOR/p70S6K signaling pathway of four weeks, and (d) the bar graph of the expression levels of the mTOR/p70S6K signaling pathway of eight weeks. All results are expressed as the mean ± SD. ^∗^Means *P* < 0.05 between diabetic group; ^#^means *P* < 0.05 between diabetic sham group.

The mTOR/p70S6K signaling pathway plays an important role in the regulation of autophagy. The phosphorylation of mTOR and p70S6K activates signaling pathways and inhibits autophagy. To investigate the mechanism of RYGB-activated autophagy, we evaluated the phosphorylated of mTOR and p70S6K by Western blot. RYGB significantly inhibited the levels of p-mTOR and p-p70S6K at two weeks postoperatively. (*P* < 0.05). At four weeks postoperatively, the phosphorylation levels of mTOR and p70S6K in liver tissue of RYGB group were observed to be significantly decreased (*P* < 0.05). At eight weeks postoperatively, the phosphorylation levels of mTOR and p70S6K in liver tissue of RYGB group were observed no significant differences (*P* > 0.05). These results indicate that RYGB activates the autophagy and promotes the lipid metabolism. It may be partially related to the inhibition of the mTOR/p70S6K signaling pathway (Figure 5).

### 3.6. GLP-1 Reduced Fat Load and Increased Fatty Acid *β*-Oxidation by Activated Autophagy in Cultured Hepatic Cells

**Figure 6:**
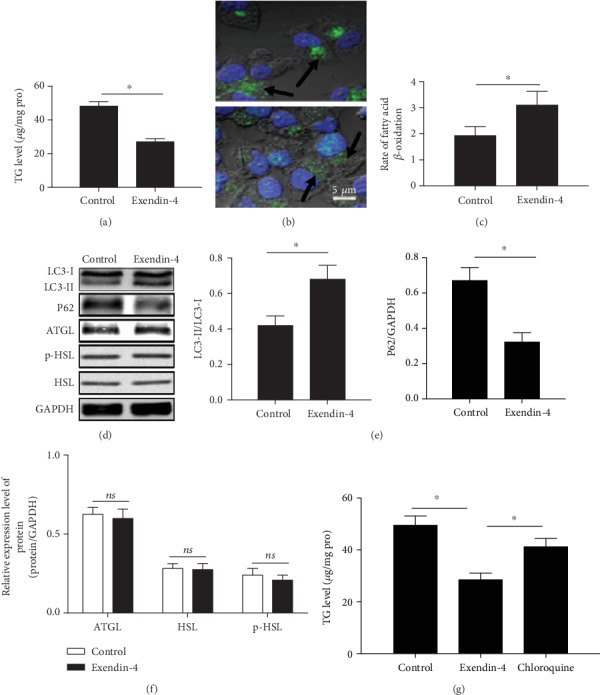
GLP-1 reduced fat load and increased fatty acid *β*-oxidation by activated autophagy in cultured hepatic cells. (a) TG levels, (b) lipid staining with BODIPY493/503 (scale bar = 5 *μ*m), (c) rate of fatty acid *β*-oxidation, (d) Western blot, (e) the bar graph of the expression levels of autophagy-related proteins, (f) the bar graph of the expression levels of cytosolic lipases, and (g) TG levels. All results are expressed as the mean ± SD. ^∗^Means *P* < 0.05 between each group.

Furthermore, we examined the effect of GLP-1 on hepatocyte autophagy *in vitro*. HepG2 cells were chosen, since they maintain many normal hepatocyte metabolic functions, and exendin-4 was used as GLP-1 analogs [26, 27]. Exendin-4 significantly decreased hepatocyte TG content in the presence of exogenous lipid supplementation with oleate (*P* < 0.05, Figure 6(a)). Consistent with the decreased TG levels, lipid staining with BODIPY493/503 revealed decreased lipid droplet numbers and size in the hepatocytes with exendin-4 (Figure 6(b)). The rate of fatty acid *β*-oxidation, indicative of the levels of FFA generated by TG hydrolysis, increased after exendin-4 treatment (*P* < 0.05, Figure 6(c)). Treatment with exendin-4 for 24 h increased LC3-II levels and decreased P62 protein expression, strongly suggesting that GLP-1 activated autophagy (Figures 6(d)–6(e)). Since intracellular TG lipolysis is considered to be catalyzed by lipid droplet lipases, we also examined the effects of exendin-4 on two important cytosolic lipases including HSL and ATGL. In contrast to the inducing effect of exendin-4 on autophagy, this medicine did not affect ATGL, p-HSL, and HSL protein expression after 24 h treatment (Figures 6(d) and 6(f)). Pharmacological inhibition of autophagy with chloroquine significantly increased hepatocyte TG content in the presence of exendin-4 (Figure 6(g)). Thus, these results suggested that GLP-1 reduced fat overload under the condition of excess of lipid burden by activating autophagy and increasing lipolysis.

### 3.7. GLP-1 Activated Autophagy *in vitro* through the mTOR/p70S6K Signaling Pathway

**Figure 7:**
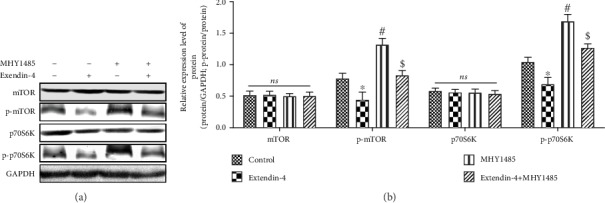
GLP-1 activated autophagy *in vitro* through the mTOR/p70S6K signaling pathway. (a) Western blot of the mTOR/p70S6K signaling pathway-related proteins, (b) the bar graph of the expression levels of the mTOR/p70S6K signaling pathway-related proteins. All results are expressed as the mean ± SD. ^∗^Means *P* < 0.05 between control group; ^#^means *P* < 0.05 between exendin-4 group; ^$^means *P* < 0.05 between MHY1485 group.

To further confirm the relationship between the autophagy activated by GLP-1 and the mTOR/p70S6K signaling pathway, we pretreated HepG2 cells with MHY1485, the mTOR1 agonist. The results showed that GLP-1 significantly reduced the phosphorylation (*P* < 0.05) of mTOR and p70S6K, while the mTOR1 agonist MHY1485 could significantly increase the phosphorylation of mTOR and p70S6K (*P* < 0.05). GLP-1 can partially reverse the function of MHY1485 to promote the role of the phosphorylation of mTOR and p70S6K (*P* < 0.05) (Figure 7). These results suggest that GLP-1 could activate autophagy by regulating mTOR/p70S6K signaling pathways and increase the fat decomposition, leading to reduce the fat under the condition of excessive lipid.

## 4. Discussion

RYGB has been reported to be effective in improving liver steatosis, inflammatory necrosis, and fibrosis [28–30]. Weiner reported a complete remission rate of 82.8% for NAFLD post RYGB in a retrospective cohort study of obese T2DM patients [31]. Caiazzo et al. reported that the degeneration, inflammation, and fibrosis in the liver were significantly improved at 1 and 5 years after RYGB surgery, and there was no difference in liver histology between 1 year and 5 years after surgery [32]. Those researches demonstrated that the triglyceride levels in the liver have decreased dramatically one week after RYGB, which rapidly and permanently reduce fat accumulation in the liver or reverse hepatic steatosis. The detailed mechanism of RYGB in reducing fat accumulation is still unclear. The results in this study indicate that in obese T2DM rats, the level of DGAT2 in the liver is significantly decreased, and the expression of p-HSL is significantly increased two weeks after RYGB. At two and four weeks postoperatively, the ratio of autophagy-related protein LC3-II/LC3-I was increased, and the expression of P62 was decreased. RYGB inhibits the fat synthesis, promotes the fat hydrolysis, rapidly decreases TG content in the liver, and reduces the insulin resistance of the liver in a short period of time, and the activation of autophagy plays a role in correcting the disorders of hepatic lipid metabolism. In addition, after RYGB, the increase of GLP-1 secretion was positively correlated with LC3-II/LC3-I ratio in the liver. The results *in vitro* suggest that GLP-1 can promote TG hydrolysis in hepatocytes by activating autophagy. Therefore, the increase of GLP-1 secretion after RYGB may be a mechanism of upregulation of autophagy activity in the liver.

The insulin resistance is generally considered as an initiating factor of NAFLD [33]. And the hepatic insulin sensitivity can be significantly improved by RYGB [34]. We proved that the insulin sensitivity index of liver in the RYGB group is higher than that in sham group at two and four weeks after surgery. Mathurin et al. found that the hepatic steatosis was significantly improved preoperatively, and the degree of IR remission was closely related to liver histology improvement [35]. Under Oil Red O staining, we showed that the volume of lipid droplets is reduced, and the number is reduced in the hepatocytes of rats after RYGB. At two weeks, four weeks, and eight weeks after operation, the TG content in the liver tissue of the RYGB group was 90% and 91% lower than that in the sham operation group, and the plasma FFA concentration was decreased by 43% and 57%. FFA levels are reduced in the postoperative RYGB, and liver fat accumulation is an important mechanism for upregulation of hepatic insulin sensitivity [36]. Compared with that at four weeks after operation, the TG content in the liver of the rats decreased slightly, and the insulin sensitivity index of liver was increased, but there was no statistical difference. We proved that the short-term decline of hepatic fat after RYGB plays a key role in the recovery of postoperative liver lipid metabolism disorders.

The disorder of fat metabolism is a major problem for T2DM patients with hepatic steatosis. The liver is the main organ to maintain the balance of fat metabolism. The imbalance of anabolism, transport, and catabolism of TG in liver cells is the main factor of fat metabolism disorder and the formation of fatty liver [37, 38]. DGAT2 is the main rate-limiting enzyme in the TG synthesis pathway and is highly expressed in both fat and liver [39]. Choi et al. administered diet-induced obese mice with the treatment of DGAT2's antisense oligonucleotide (ASO) to inhibit DGAT2 expression and found that fatty liver and hyperlipidemia were significantly improved [40]. McLaren et al. found that the TG content in DGAT2 overexpressing mice increased, and the secretion of VLDL was not affected [41]. Microsomal triglyceride transfer proteins (MTP) are mainly distributed in hepatocytes and intestinal epithelial cells, which are key lipid transporters involved VLDL assembly and secretion of TG in the liver. Chang et al. confirmed that in high-fat diet-fed NAFLD rats, the promoter of MTP in the rats' liver was highly methylated, leading to the inhibition of MTP mRNA and protein, the disorders of TG secretion, and the deposition of hepatic lipid [42]. ATGL and HSL are key hydrolases in mobilization of body fat. Mice with ATGL gene silencing not only increased the total amount of TG in adipose tissue, but also increased the TG content in other nonadipose tissues including the liver [43]. Reid et al. reported that the overexpressing HSL and ATGL in adenovirus vector can reduce TG content of liver in ob/ob mice and high fat diet-induced obese mice by 40% to 60% [44]. Our results showed that compared with rats in sham group, the expression of DGAT2 in the liver of the RYGB was significantly decreased, the expression of p-HSL protein was significantly increased, and the expression of MTP protein was also increased, but the difference was not statistically significant. RYGB inhibited hepatic TG synthesis and stimulated hepatic TG hydrolysis in the short term, but RYGP had no effect on hepatic TG secretion. The expression of key enzymes of DGAT2, P-HSL, ATGL, and MTP in hepatic lipid metabolism at four and eight weeks after surgery was not different between the diabetic groups. We believe that RYGB surgery can rapidly reduce the accumulation of fat in the liver by rapidly inhibiting local fat synthesis in the liver and upregulating local mobilization of liver fat.

Autophagy is a process in which cells degrade damaged organelles and macromolecules dependent on lysosomes. Autophagy is mediated by organelles such as autophagosomes and lysosomes and is involved by more than 30 related proteins. Among them, LC3 is considered to be an autophagosome's marker, and the increase of LC3-II/LC3-I ratio represents the activation of autophagy [45]. P62 can transport proteins with ubiquitination modification to autophagosomes, and P62 aggregation increased as autophagy is inhibited [46]. Kwanten et al. found that in NAFLD patients, the ratio of LC3-II/LC3-I in the liver decreased, and the expression of P62 protein increased, and the ratio of LC3-II/LC3-I decreased following the severity of hepatic steatosis [47]. The distribution of lipid droplets was negatively correlated with the distribution of autophagosomes, suggesting that the level of autophagy is closely related to the development of NAFLD. The results suggested that the ratio of LC3-II/LC3-I of rats in the RYGB is significantly higher than that in the sham and the diabetic group at two and four weeks after surgery, and the expression of P62 protein is significantly decreased, suggesting that RYGB activates autophagy in the liver. These reports are consistent with our research. The activation of hepatic autophagy after RYGB may play an important role in the rapidly declination of hepatic lipids in short time and the reduction of hepatic lipid accumulation in long time.

mTOR is an important signaling molecule family in autophagy signaling pathway. mTOR collects and transmits multiple autophagy signals upstream and negatively regulates the autophagy process. The activated mTOR complex mainly regulates the downstream target molecule through phosphorylation, p70S6K is one of its target molecules, and the p70S6K of phosphorylation is the marker of mTOR activation [48].

Studies have shown that many obese patients with disordered lipid metabolism have dysfunction of mTOR in the body, suggesting that mTOR is involved in lipid metabolism [49–51]. Studies have shown that mTOR can increase the expression of SREBPs in fat synthesis-related genes and promote the splicing modification of SREBPs in Golgi apparatus. As mTORC1 or raptor is silenced, the expression of fat synthesis-related protein, the sterol responsive element binding protein 1 (SREBP1), and its target genes Fasn and Acly are inhibited [52]. Through the analysis of the downstream factors, we found that silencing p70S6K can cause a significant decrease in the expression of SREBPs. It was reported that p70S6K, the mTOR downstream factor, plays an important role in regulating the transcriptional regulation of SREBP-1, and mTOR can also regulate 4EBP1, the downstream factor of SREBP-1 to control the lipid metabolism [53]. The occurrence of obesity was prevented, if p70S6K or 4EBP1t was inhibited [54]. This study also showed that the phosphorylation levels of mTOR and p70S6K protein of liver in RYGB group were also significantly decreased at two and four weeks after surgery. This indicates that RYGB can activate autophagy, and promote lipid metabolism, which may be dependent on the inhibition of mTOR/p70S6K signaling pathway.

GLP-1 is a peptide hormone synthesized by the ileum end and colon L cells. Bernsmeier et al. showed that the secretion of GLP-1 in patients with NAFLD was significantly lower than that in normal subjects, suggesting that GLP-1 secretion is impaired in patients with NAFLD [55]. In obese patients with RYGB, hepatic degeneration is relieved, and the GLP-1 secretion is increased as the remodels of the intestinal structure during RYGB [56]. This study suggested that plasma GLP-1 levels have increased significantly at two weeks after RYGB. After treatment with obese mice with GLP-1 analog exendin-4 for four weeks, Sharma et al. found that the formation of autophagy lysosomes increased, the expression of autophagy-related proteins increased, and reduced liver damage caused by lipid deposition [57]. Based on this study, we performed the correlation analysis with plasma GLP-1 levels and the ratio of LC3-II/LC3-I in the liver. The results showed that LC3-II/LC3-I levels are significantly positively correlated with plasma GLP-1 concentrations. It is speculated that the activation of autophagy may be associated with increased GLP-1 secretion after RYGB.

GLP-1 receptors are present on hepatocytes, and the exposure of hepatocytes to GLP-1 receptor agonists led to a reduction of fat load in hepatocytes [58]. The results *in vitro* showed that GLP-1 analogs reduced hepatocyte fat overload under the condition of excess of lipid burden by activating autophagy and increasing lipolysis. Pharmacological inhibition of autophagy with chloroquine significantly increased the hepatocyte triglyceride content in the presence of GLP-1 analogs. In order to further explore the role of mTOR/p70S6K signaling pathway in autophagy activated by GLP-1, we injected the mTOR1 agonists MHY1485 into rats and found that GLP-1 can partially reverse the increase of mTOR and p70S6K protein phosphorylation levels, which suggested that GLP-1 activated autophagy and increased the fat decomposition by regulating the mTOR/p70S6K signaling pathway, thereby regulating the fat overload under the condition of excess of lipid burden.

The results confirmed that RYGB can quickly reduce liver fat accumulation through the regulation of liver fat metabolism pathway. The activation of autophagy by RYGB will play a role in the mitigation of hepatic lipid toxicity in the short and long term. The increase of postoperative GLP-1 secretion may be related with the increase of the activity of liver autophagy. In recent years, the effect of RYGB on the nervous system has become a research hotspot. The central nervous system, especially the hypothalamus, can control the central and peripheral energy homeostasis through neuropeptides and neurotransmitters [59]. Some studies have confirmed that changes in the nervous system can be significantly observed after RYGB [60–62]. Liver fat metabolism is regulated by not only humoral but also the nervous system. The effect of RYGB on neurotransmitters in hepatic tissue and the effects of postoperative nervous system on hepatic lipid metabolism have not been reported; thus, we will focus on this research area.

## 5. Conclusion

We investigated the therapeutic function of RYGB on hepatic lipid metabolism in obese T2DM rats and found that RYGB could rapidly reduce hepatic lipid toxicity and improve insulin sensitivity through activating the hepatic fat hydrolysis pathway and inhibiting the liver fat synthesis pathway. The increased hepatic autophagy activity post RYGB played an important role in the long-term maintenance of hepatic lipid metabolism balance, which may be related to the inhibition of mTOR/p70S6K signaling pathway.

## Data Availability

The datasets used and analyzed during the current study are available from the corresponding author upon reasonable request.
